# Ironing process optimization for enhanced properties in material extrusion technology using Box–Behnken Design

**DOI:** 10.1038/s41598-024-52827-5

**Published:** 2024-01-27

**Authors:** Hussein Alzyod, Peter Ficzere

**Affiliations:** Department of Railway Vehicles and Vehicle System Analysis, Faculty of Transportation Engineering and Vehicle Engineering, Budapest University of Technology and Economics, Műegyetem Rkp. 3, 1111 Budapest, Hungary

**Keywords:** Materials science, Mechanical engineering

## Abstract

Material Extrusion (MEX) technology, a prominent process in the field of additive manufacturing (AM), has witnessed significant growth in recent years. The continuous quest for enhanced material properties and refined surface quality has led to the exploration of post-processing techniques. In this study, we delve into the ironing process as a vital processing step, focusing on the optimization of its parameters through the application of Design of Experiments (DoE), specifically the Box–Behnken Design (BBD). Through a systematic examination of ironing process parameters, we identified optimal conditions that resulted in a substantial reduction in surface roughness (Ra) by approximately 69%. Moreover, the integration of optimized ironing process parameters led to remarkable improvements in mechanical properties. For instance, the Ultimate Tensile Strength (UTS) saw a substantial improvement of approximately 29%, while the compressive strength (CS) showed an increase of about 25%. The flexural strength (FS) witnessed a notable enhancement of around 35%, and the impact strength (IS) experienced a significant boost of about 162%. The introduction of ironing minimizes voids, enhances layer bonding, and reduces surface irregularities, resulting in components that not only exhibit exceptional mechanical performance but also possess refined aesthetics. This research sheds light on the transformative potential of precision experimentation, post-processing techniques, and statistical methodologies in advancing Material Extrusion technology. The findings offer practical implications for industries requiring high-performance components with structural integrity and aesthetic appeal.

## Introduction

Material extrusion (MEX), which is also recognized as Fused Filament Fabrication (FFF) or as Fused Deposition Modeling (FDM)^1,2^, is an additive manufacturing (AM) process that employs a heated extruding nozzle to deposit a continuous filament of thermoplastic or polymer material layer by layer to create 3D parts^3,4^. MEX technology has several advantages over other AM technologies, including the diverse choice of filaments (such as acrylonitrile butadiene styrene (ABS), polylactic acid (PLA), acrylonitrile styrene acrylate (ASA), polyether ether ketone (PEEK), Polypropylene (PP) ^5^, and polyethene terephthalate glycol (PETG) ^6^, etc.), a wide variety of filament colours ^7,8^, fibre and metal reinforced polymer matrix composites with multi-functional properties ^9,10^, ease of material loading and replacement, capability to utilize recycled materials ^11–13^, inexpensive maintenance rates, rapid manufacturing of small parts, low tolerance, ability to print complex geometries ^14^, and safe and nontoxic ingredients ^15,16^. These advantages make it a popular manufacturing method in various industries ^17^. As a result, there is a growing need to focus on developing functional and sustainable products in industrial sectors such as medical ^18,19^, automotive^20–22^, aviation^23^, and fashion ^24^. However, MEX has some challenges, including anisotropic properties ^25^, limited shape accuracy^26,27^, and varying surface roughness in flat and inclined surfaces ^28^. However, it is worth noting that MEX technology has some limitations. For instance, accuracy can be compromised, especially when support structures are required for a MEX geometry featuring an overhang exceeding 45° or protruding structures ^29^. Additionally, gaps between toolpaths, prolonged printing times for thinner layers, and thermal gradients during printing may lead to issues such as delamination or warping. These problems, in turn, result in stresses within printed components and contribute to a rough surface finish, impacting the overall quality of the final product. Research efforts to address these challenges and expand the applications of MEX are, therefore, increasingly crucial ^30^. The properties of printed products depend on the used material and the pre-processing parameters, such as printing speed, layer thickness, printing temperature, bed temperature, raster angle, toolpath, printing orientation, etc. ^31^. Extensive research has been done to investigate and optimize these pre-processing parameters to improve the mechanical properties of MEX parts ^32–36^. Kumar et al. ^37^ made significant improvements in addressing challenges in MEX. Through a meticulous study involving Taguchi optimization and CRITIC-embedded WASPAS, they optimized layer thickness, print speed, and temperature, resulting in substantially improved mechanical attributes. The research demonstrated enhanced strength and minimized defects, offering valuable insights for advancing AM methodologies. Some researchers also used post-processing techniques to improve the mechanical properties of MEX parts. Chen ^38^ used laser polishing post-processing to enhance the properties of Al-PLA composite samples made using FDM. The results showed that laser polishing decreased the porosity inside the FDM parts and improved interfacial adhesion between the Al fibres and PLA matrix. This led to enhanced dynamic mechanical properties and an improvement in tensile strength and Young's modulus. Beniak et al. ^39^ employed acetone vapour post-processing to enhance the surface quality and compressive strength of FDM parts made of ABS material. The smoothing process resulted in a 97.63% decrease in surface roughness, and the compressive strength of the specimen increased by more than 21% when exposed to 5 min of acetone vapour. Kalyan et al. ^40^ increased the surface quality and hardness of ABS-FDM samples using vapour smoothing post-process. Hambali et al. ^41^ implemented chemical treatment as a post-processing methodology to improve the surface quality of FDM-ABS components. The findings revealed a significant enhancement of about 97% in surface roughness, simultaneous with a noticeable reduction of around 43% in tensile strength. Ironing is a simple and inexpensive post-processing technique that can be performed using any typical FDM printer. Despite its potential benefits, there have been relatively few studies on the effects of ironing with default parameter settings on FDM part properties. Sardinha et al. ^42^ investigated the influence of ironing post-processing on ABS parts produced employing the FDM technique. The study concluded that surface roughness and warpage deformation were reduced by up to 60% and 30%, respectively, while Griffiths ^43^ investigated the impact of subtractive machining, ironing, and burnishing on the conductivity of inkjet-printed silver nano-particle ink on FDM printed PLA parts and concluded that ironing enhanced conductivity by 72%. Sardinha et al. ^44^ also demonstrated that ironing can reduce distortion levels by up to 33% and surface roughness by up to 60% in ABS samples produced using FDM technology. In another investigation, Paz et al. ^45^ studied the impact of ironing and two other post-processing techniques on the electrical conductivity of GNP-reinforced nanocomposite ABS parts produced using FDM. The study concluded that ironing resulted in significant improvements in the electrical characteristics of the part surface. Butt ^46^ studied the effect of ironing post-processing on the surface roughness, dimensional accuracy, and hardness of ABS and ASA FFF-printed parts. The results showed that surface roughness, dimensional accuracy, and hardness were improved in both ABS and ASA.

It is widely recognized that post-processing techniques play a crucial role in enhancing the mechanical properties of MEX parts by improving the surface quality^47^. However, in this study, we aim to study the effect of using the ironing post-processing technique after every printed layer on the mechanical properties of PLA components made utilizing the MEX process. Ironing has shown promising results in improving surface quality by reducing surface roughness and decreasing the visibility of layer lines^48^. By performing ironing as a processing step, the aim is to improve the adhesion between layers and enhance the mechanical properties of the part by reducing the occurrence of interlayer voids and delamination. As with any processing technique, ironing processing involves controllable parameters, specifically spacing between passes, path speed, and flow rate. In this particular study, the impact of these ironing process parameters on a range of responses, including ultimate tensile strength (UTS), compressive strength (CS), flexural strength (FS), impact strength (IS), and surface roughness (Ra), was investigated using Box–Behnken Design (BBD). This study aims to thoroughly investigate the influence of MEX ironing process parameters on the mechanical and surface properties of printed parts. By conducting a comprehensive analysis and validation study, we seek to provide valuable insights that will contribute to the optimization of the MEX process, ultimately enabling the production of high-quality parts with enhanced mechanical performance and surface characteristics.

## Materials and methods

### Material, samples, and printing parameters used

**Figure 1:**
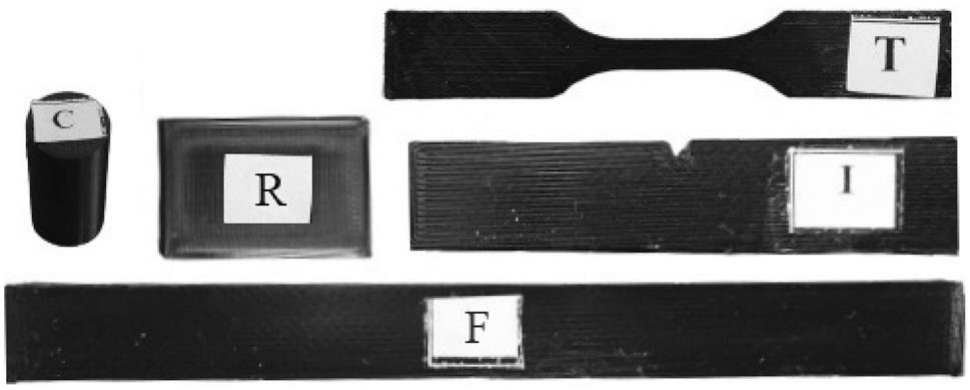
Printed samples: *T* tensile, *C* compressive, *F* flexural, *I* impact, *R* roughness.

Black colour EcoPLA filament, a nontoxic, biodegradable 3D printing material with 1.75 mm diameter and ± 0.05 mm tolerance, sourced from the supplier 3Djake^49,50^. The process of creating a 3D model for the specimens began with the utilization of SolidWorks TM. After that, the geometry was exported as a .stl file to import it to the PrusaSlicer software, which is compatible with a wide range of 3D printers and enables the application of ironing processing. This facilitated the conversion of the .stl file into G-code. All printed specimens were printed utilizing an open-source printer, Creality Ender3-V2 printer. A 30 mm × 20 mm × 2 mm sample was used for the roughness measurements. For the mechanical properties testing, all the samples were prepared according to the American Society for Testing and Materials (ASTM) standards. Specifically, D638 type V was used for the tensile test^51^, D695-15 for the compressive test^52^, D256 for the impact test^53^, and D790-17 for the flexural test^54^. Tensile tests were conducted on a ZwickiLine machine at a crosshead speed of 5 mm/min, compression tests were performed on a Zwick Z020 machine at a constant compression rate of 2 mm/min, impact tests were carried out on a Resil 5.5 machine with an energy level of 4 Joules, and flexural tests were executed on a Zwick/Roell machine with a span width of 40 mm. To unravel the intricate details concealed within the material matrix, a meticulous microscopic analysis for UTS, compressive strength, flexural strength, and impact strength was conducted using a Dino-Lite microscope, while the surface roughness of the uppermost surface of the MEX specimens was measured using Keyence VR-5000. The surface roughness of PLA-printed parts was assessed using optical interferometry. A high-magnification camera, capable of up to 160 × magnification, was employed for detailed examination. Surface roughness values ranging from 0.1 to 2 µm were measured using a cut-off length/sampling length of 0.8 mm/4 mm, while values ranging from 2 to 10 µm were measured with a cut-off length/sampling length of 2.5 mm/12.5 mm^55^. The measurement system provided a display resolution of 0.1 µm, ensuring precision in the evaluation of surface characteristics. As the aim of this study is to investigate the ironing parameters, the printing parameters were set, as shown in Table 1. The selection parameters' values were based on literature and the material data sheet^49,56–58^, and Fig. 1 depicts the printed test samples.

**Table 1:** Set printing parameters for the printed parts.

Printing parameter	Value	Unit
Printing rate	60	mm/s
Layer height	0.2	mm
Extruder head	0.4	mm
Printing temperature	205	°C
Bed temperature	50	°C
Printing density	100	%
Infill pattern	Line	
Build orientation	XYZ	
Print direction	Horizontal

### Optimization of the ironing parameters through the utilization of Box–Behnken Design (BBD)

#### Design of experiments (DoE)

The mathematical methodology known as Design of Experiments (DOE) is employed for the systematic planning, execution, and analysis of experiments, as well as the interpretation of data derived from these experiments^59^. This field, rooted in applied statistics, plays a crucial role in the scientific investigation of systems, aiming to enhance product quality and reliability^60^. There are many types of DoE used to study the impact of process parameters in MEX, such as Taguchi's orthogonal design^61,62^, BBD, Response Surface Methodology (RSM)^63^, and Central Composite Design (CCD)^64^. In this investigation, we used DoE, specifically BBD, to advance our understanding of the intricate relationships between printing parameters and material properties in the realm of MEX technology. BBD is a type of experimental design used in statistical analysis and modelling. It is a response surface methodology that is often used to optimize process parameters^65,66^.

BBD employed in this study follows a three-level factorial design approach, where the factors under investigation are examined at low, medium, and high levels^67,68^. This design is constructed using a series of points that form a three-level factorial design, which is then augmented with additional points forming a central composite design. The central composite design allows for the estimation of the curvature of the response surface in the vicinity of the optimal factor settings^69^. One notable advantage of the BBD is its ability to achieve meaningful results with fewer experimental runs compared to a full factorial design, leading to enhanced efficiency and cost-effectiveness^70^. Moreover, the BBD provides a reliable approximation of the response surface within the area of interest, making it a valuable tool for optimization. Several researchers have effectively utilized the BBD to examine the impact of various processing parameters on the mechanical properties of printed parts. Mohan^71^ used BBD to investigate the effects of three printing parameters, including infill density, layer thickness, and bed temperature, on micro-hardness, density, print time, and surface roughness of ABS parts made using the FDM process. Vardhan^72^ studied the impact of layer height and build orientation on the tensile strength of FFF parts made of ZP150 powder using the BBD approach. The study found that layer thickness has a negative effect on tensile strength, meaning that increasing layer thickness decreases tensile strength. The orientation of the part also has an impact on tensile strength. When the part is oriented in the XY plane, increasing rotation leads to an increase in tensile strength. However, for the XZ and YZ planes, tensile strength decreases until reaching an angle of 45°, after which it rapidly increases up to 90°. Prabakaran^73^ used BBD to investigate the influence of raster angle, printing temperature, and layer thickness on tensile strength, fracture strain, and elastic modulus of ASA parts fabricated using the FDM technique. The conducted study found that the layer thickness has a greater impact on these properties compared to the raster angle and printing temperature.

#### BBD methodology 

**Figure 2:**
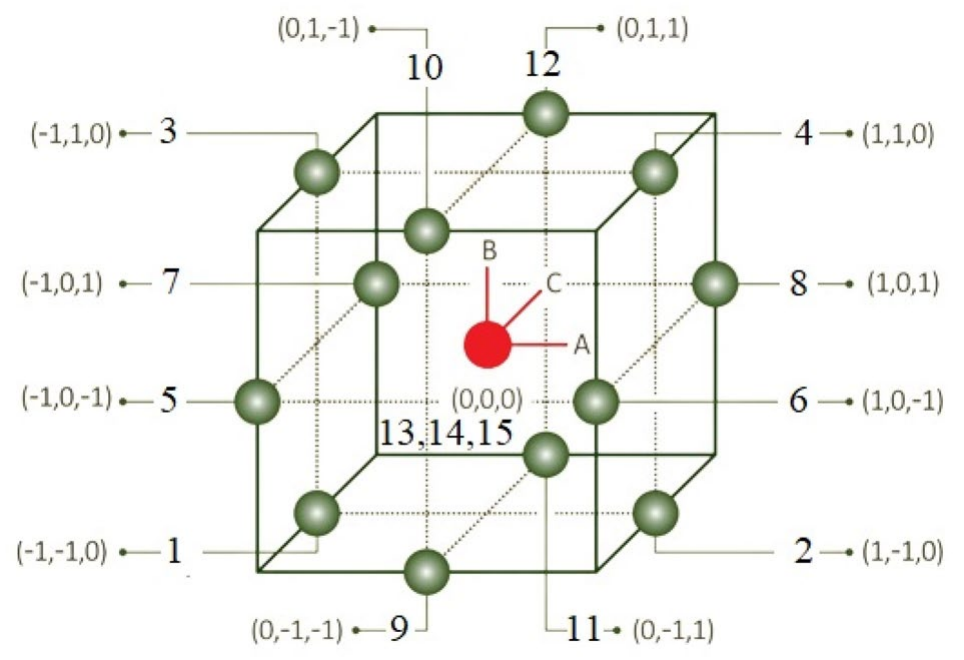
Box–Behnken Design of design points.

**Figure 3:**
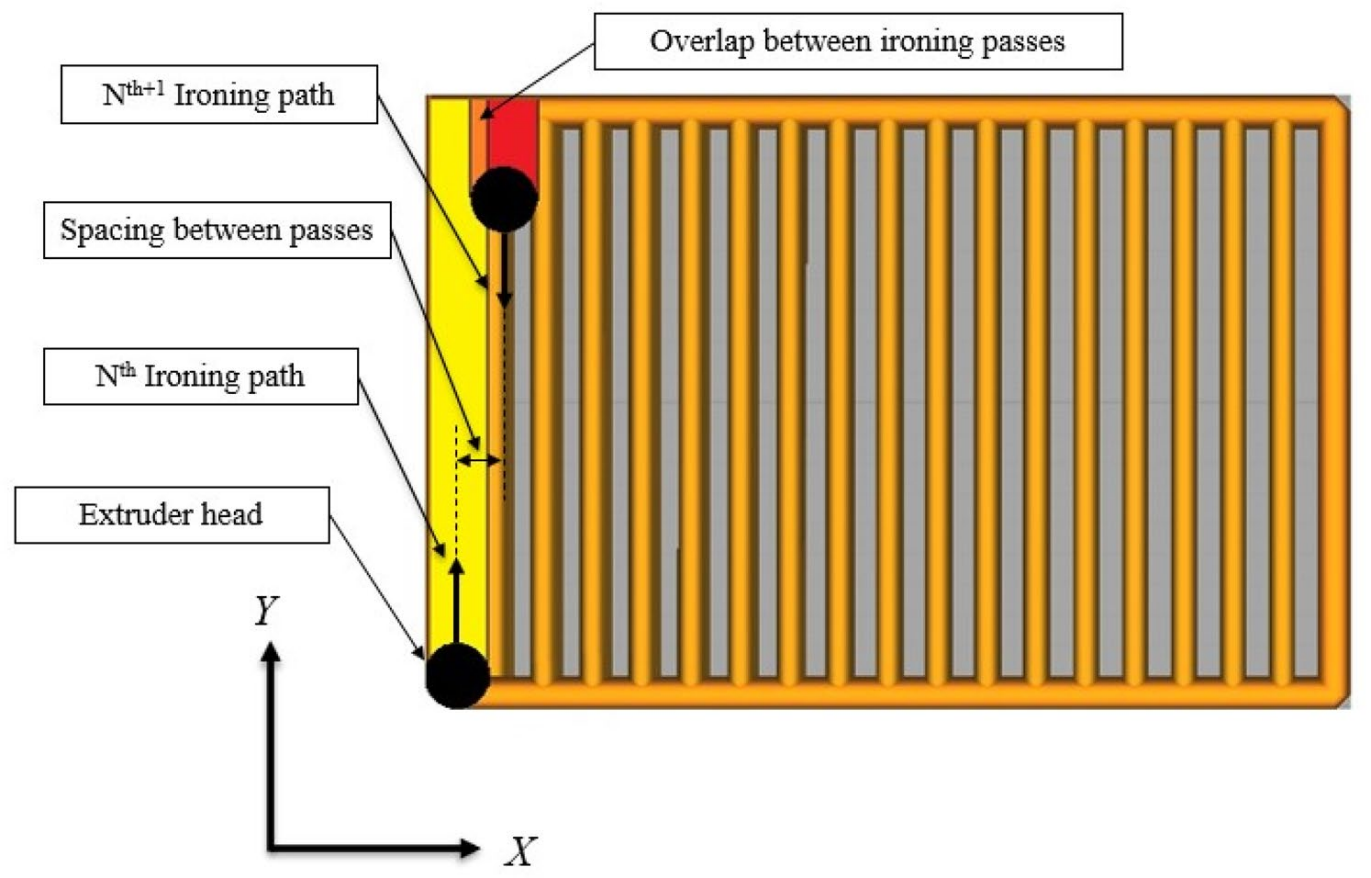
Visual representation of the spacing between passes.

The most commonly used BBD array is the L15, which involves conducting fifteen experiments with three centre-point repetitions^74^. A graphical representation of the BBD combinations is shown in Fig. 2, which illustrates a cube with the centre point (0,0,0) and other possible combinations expressed as points with three coordinates located on the cube facets and edges. It is important to note that BBD only considers the midpoints of the edges and the centre point, which translates into thirteen points: twelve edge points plus the centre point. Furthermore, the centre point is replicated three times, which results in a total of fifteen experiments^75^. By repeating the centre points three times, we can estimate the pure error of the experimental process and use this information to assess the significance of any observed effects. This also helps to increase the precision and reliability of the estimates for the parameters being investigated. The three ironing parameters, spacing between passes, path speed, and flow rate, were used as ironing process parameters with the three levels, as shown in Table 2. These levels were selected based on the PrusaSlicer software recommendations. "Spacing between passes" refers to the distance between successive ironing paths, which is influenced by the extruder head diameter. Increasing the spacing between passes will result in a reduction in the overlap between ironing passes, and Fig. 3 provides a visual representation of the spacing between passes. The scheme "Path speed" is the speed of the heated nozzle movement over the deposited layer. "Flow rate" refers to the amount of filament that is deposited during the ironing process, expressed as a percentage of the set layer thickness. The primary objective of this experimental work is to utilize analysis of variance (ANOVA), microscopic analysis of the internal structure, and contour plots to determine the significant contribution of the ironing process parameters to the responses.

**Table 2:** BBD parameter design.

Ironing parameter	Codes	Levels		
		− 1	0	1
Spacing between passes (mm)	A	0.1	0.2	0.3
Path speed (mm/s)	B	10	20	30
Flow rate (%)	C	10	15	20

## Results and discussion

The application of the BBD coupled with the ironing process has demonstrated its efficacy in achieving a significant enhancement in mechanical properties and surface roughness of the MEX parts. The UTS increased to 56.47 MPa, marking a notable improvement of approximately 28.6% from the initial 43.91 MPa. Similarly, the compressive strength exhibited substantial growth, rising from 62.7 to 78.34 MPa, representing an enhancement of approximately 24.9%. The flexural strength also demonstrated a notable increase, reaching 85.73 MPa, which signifies a considerable improvement of approximately 35.4% compared to the initial 63.34 MPa. Additionally, the impact strength saw a significant boost, measuring 9.84 kJ/m^2^ after ironing, an impressive improvement of approximately 161.7% compared to the initial 3.76 kJ/m^2^. Furthermore, the surface roughness Ra experienced a substantial reduction, measuring only 2.73 μm after ironing compared to the initial 8.829 μm, representing a reduction of approximately 69.1%. These results underscore the significant impact of the ironing process on enhancing the mechanical properties and surface quality of MEX parts, and they demonstrate the potential for optimizing printing parameters and post-processing techniques in the AM industry. Table 3 presents 15 experimental sets and their results with varying process parameters, which were formulated using the statistical software MINITAB 21. Each result in the table represents the mean value obtained from three measurements. While the ironing process has shown significant benefits in enhancing mechanical properties and surface quality, it is essential to acknowledge potential drawbacks. One consideration is the potential increase in printing time associated with the ironing step. Additionally, the impact on the roughness of the side surface warrants discussion, and future research could delve into optimizing ironing parameters to mitigate any adverse effects on printing time and side surface quality.

**Table 3:** BBD experimental design and the output results with the standard deviations (SD).

Run no	A	B	C	UTS ± SD (MPa)	CS ± SD (MPa)	FS ± SD (MPa)	IS ± SD (kJ/m^2^)	Ra ± SD (µm)
1	− 1	− 1	0	51.16 ± 2.25	72.7 ± 2.5	81.44 ± 1.43	9.46 ± 0.55	2.993 ± 0.745
2	1	− 1	0	49.77 ± 3.07	68.22 ± 2.0	79.91 ± 0.55	9.14 ± 0.59	5.682 ± 0.912
3	− 1	1	0	55.36 ± 3.09	74.74 ± 2.3	82.57 ± 1.06	9.45 ± 0.43	3.302 ± 0.461
4	1	1	0	50.93 ± 2.69	67.2 ± 2.4	80.23 ± 1.22	8.86 ± 0.59	5.468 ± 0.582
5	− 1	0	− 1	54.56 ± 2.19	76.94 ± 2.7	83.68 ± 1.08	9.81 ± 0.56	2.731 ± 0.602
6	1	0	− 1	45.25 ± 2.88	71.37 ± 2.1	80.54 ± 0.95	9.07 ± 0.44	5.935 ± 1.028
7	− 1	0	1	54.60 ± 2.49	75.83 ± 2.3	82.88 ± 1.12	9.52 ± 0.38	3.357 ± 0.715
8	1	0	1	43.68 ± 3.07	70.49 ± 2.0	80.77 ± 0.28	8.77 ± 0.58	5.786 ± 0.725
9	0	− 1	− 1	54.29 ± 2.21	76.86 ± 2.2	82.12 ± 0.55	9.62 ± 0.58	4.629 ± 0.315
10	0	1	− 1	47.73 ± 3.22	76.61 ± 2.1	81.35 ± 0.65	9.09 ± 0.53	4.727 ± 0.358
11	0	− 1	1	51.11 ± 3.37	78.34 ± 1.8	83.00 ± 0.67	9.16 ± 0.41	4.603 ± 0.507
12	0	1	1	55.85 ± 2.95	77.26 ± 2.1	82.99 ± 0.54	9.84 ± 0.08	4.762 ± 0.525
13	0	0	0	52.30 ± 2.82	76.77 ± 1.8	83.11 ± 0.61	9.63 ± 0.47	4.086 ± 0.041
14	0	0	0	53.92 ± 3.06	76.47 ± 2.0	85.73 ± 1.25	9.76 ± 0.25	4.148 ± 0.075
15	0	0	0	56.47 ± 3.41	73.89 ± 2.5	83.85 ± 1.51	9.13 ± 0.31	3.994 ± 0.417
Without ironing				43.91 ± 1.93	62.7 ± 3.5	63.34 ± 7.37	3.76 ± 0.33	62.7 ± 4.5

### Analysis of the ironing process parameters for UTS, compressive, flexural, and impact strength, and surface roughness of PLA

To evaluate the mechanical properties of processed PLA parts, the ironing process parameters of MEX played a significant role in their fabrication. As such, we analyzed the effects of these parameters on various responses, including UTS, compressive strength, flexural strength, impact strength, and surface roughness of PLA using BBD response surface methodology. Through this analysis, we were able to obtain optimized values for the ironing process parameters of MEX.

#### Analysis of variance (ANOVA) study

In this study, we utilized an analysis of variance (ANOVA) approach to establish the relationship between the ironing process parameters and their respective contributions to the response variable, effectively identifying the most influential factors. The contribution percentages of each ironing process parameter towards the responses were determined by calculating the ratio between the total adjusted sum of squares and the individual adjusted sum of squares. This was done for both linear (A, B, and C), quadratic (AA, BB, and CC), and 2-way interaction terms (AB, AC, and BC), where A represents the spacing between passes (mm), B stands for path speed (mm/s), and C corresponds to flow rate (%). The ANOVA results provided contribution percentage insights for UTS, CS, FS, IS, and Ra, as presented in Table 4. Typically, the Fisher value 'F' was employed to identify significant contributors to the response, with a threshold of 'F' value exceeding 4^76^.

**Table 4:** Comparison of ANOVA response results.

Source	DF	F-value					P-value					Contribution percentage (%)				
		UTS	CS	FS	IS	Ra	UTS	CS	FS	IS	Ra	UTS	CS	FS	IS	Ra
Model	9	1.94	11.31	2.87	1.83	68.82	0.24	0.01	0.13	0.26	0.00	77.71	95.32	83.77	76.70	99.20
Linear	3	3.15	13.73	3.18	3.15	194.30	0.12	0.01	0.12	0.13	0.00	42.14	38.57	31.00	43.98	93.36
A	1	9.12	41.18	9.09	9.27	581.01	0.03	0.00	0.03	0.03	0.00	40.68*	38.56*	29.49*	43.18*	93.06*
B	1	0.17	0.01	0.05	0.03	0.65	0.70	0.93	0.84	0.87	0.46	0.75	0.01	0.16	0.15	0.10
C	1	0.16	0.00	0.42	0.14	1.25	0.71	0.97	0.55	0.72	0.32	0.70	0.00	1.35	0.65	0.20
Square	3	1.41	19.68	5.25	0.72	9.06	0.34	0.00	0.05	0.58	0.02	18.83	55.27	51.14	10.12	4.35
AA	1	2.63	42.83	10.41	2.02	0.13	0.17	0.00	0.02	0.21	0.74	10.59	42.67*	29.29*	9.01	0.01
BB	1	0.01	1.11	6.31	0.24	10.28	0.93	0.34	0.05	0.65	0.02	0.17	1.63	19.56*	1.10	1.33
CC	1	1.81	11.71	0.71	0.00	18.85	0.24	0.02	0.44	0.96	0.01	8.06	10.97*	2.29	0.01	3.02*
2-way interaction	3	1.25	0.53	0.17	1.62	3.09	0.39	0.68	0.91	0.30	0.13	16.74	1.48	1.63	22.60	1.49
AB	1	0.25	1.47	0.14	0.23	2.89	0.64	0.28	0.72	0.65	0.15	1.10	1.37	0.46	1.08	0.46
AC	1	0.07	0.01	0.23	0.00	6.34	0.80	0.93	0.65	1.00	0.05	0.31	0.01	0.75	0.00	1.02*
BC	1	3.44	0.11	0.13	4.62	0.04	0.12	0.76	0.74	0.08	0.85	15.32	0.10	0.42	21.52	0.01
Error	5											22.29	4.68	16.23	23.30	0.80
Total	14															

*Statistically significant results.

While the statistical analysis provided valuable insights, we recognize the importance of elucidating the underlying mechanisms driving these observed effects. The spacing between passes plays a pivotal role in determining the UTS of the PLA sample, contributing significantly with a value of 40.68%. This can be correlated with the interlayer bonding strength, where optimal spacing promotes enhanced adhesion between successive layers, resulting in improved tensile strength. When considering compressive strength, the key contributors are the spacing between passes, the square of spacing between passes, and the square of flow rate, contributing substantially at 38.56%, 42.67%, and 10.97%, respectively. These relationships could be associated with the density and arrangement of infill structures, where the optimal spacing and flow rate contribute to a more uniform and dense internal structure, consequently improving compressive strength. When considering flexural strength, the major influencers are the spacing between passes, the square of spacing between passes, and the path speed, contributing notably at 29.49%, 29.29%, and 19.56%, respectively. These factors may affect the interlayer adhesion and filament alignment, influencing the material's ability to withstand bending forces. Similarly, spacing between passes emerges as a dominant factor in determining impact strength, with a significant contribution of 43.18%. This can be correlated with the optimized infill patterns and layer bonding achieved at specific spacing values, enhancing the material's resistance to sudden impact. Finally, the primary predictors for surface roughness are the spacing between passes, the square of flow rate, and the interaction between spacing between passes and flow rate, collectively contributing a substantial 93.09%, 3.02%, and 1.02%, respectively. These parameters influence the layer-to-layer uniformity and surface irregularities, showcasing the importance of precise control over spacing and flow rate for achieving a smoother surface finish.

#### Contour plot for responses

**Figure 4:**
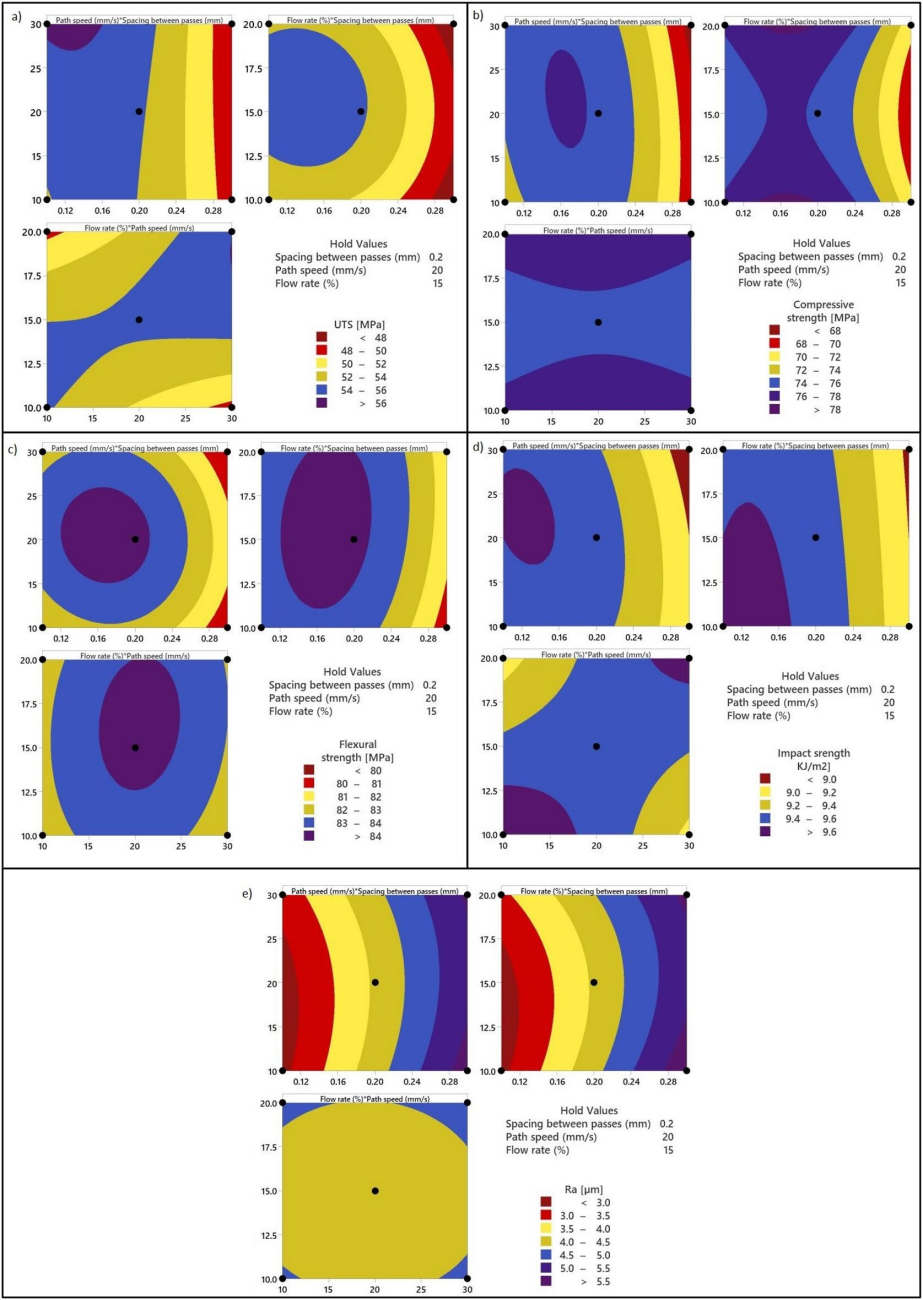
Contour plot for responses: (**a**) UTS, (**b**) CS, (**c**) FS, (**d**) IS, and (**e**) Ra.

In order to evaluate and confirm the synergistic effects of the ironing process parameters of MEX on key response variables such as UTS, compressive strength, flexural strength, impact strength, and surface roughness, a contour plot was generated. This contour plot was produced for fixed values of path spacing (0.2 mm), path speed (20 mm/s), and flow rate (15%) using MINITAB statistical software version 21, as depicted in Fig. 4. The contour plot is derived from the regression equation, aiding in the comprehensive assessment of the parameter interactions. Analyzing the UTS contour plot, as depicted in Fig. 4a, reveals distinct trends. It is evident that UTS exhibits a decrease with an increase in the spacing between passes while showing an upward trajectory as path speed is elevated. Moreover, UTS experiences a rise with an increase in the flow rate, with the highest UTS value observed at a flow rate of 20%. Notably, UTS demonstrates an inverse correlation with the interaction between path speed and flow rate. In reference to compressive strength, as elucidated in Fig. 4b, the findings are discernible. Notably, the compressive strength exhibits a diminishing trend as the spacing between passes increases. Additionally, an initial ascent in compressive strength is observed with an increase in the flow rate, although a marginal decline follows this. Furthermore, it is noteworthy that the interaction between flow rate and path speed appears to have an insignificant impact on compressive strength. In the context of flexural strength, as depicted in Fig. 4c, a distinct pattern emerges from the contour plots. It is evident that flexural strength demonstrates an initial increase, followed by a subsequent decrease, as all three parameters vary. Significantly, the peak of flexural strength is pinpointed at the centre of the contour plot, characterized by a spacing of 0.2 mm, a path speed of 20 mm/s, and a flow rate of 15%. Within the realm of impact strength, as depicted in Fig. 4d, a noticeable observation appears obviously. It becomes apparent that the interaction between flow rate and path speed has a negligible impact on impact strength. In addition, there is a notable decrease in impact strength with the growth of spacing between passes.

Concluding our analysis, Fig. 4e provides insight through contour plots depicting surface roughness. Notably, an increase in the spacing between passes correlates with an elevation in surface roughness. Conversely, an increase in both flow rate and path speed is linked to a reduction in surface roughness. It is significant to observe that surface roughness reaches its peak at the extreme levels of spacing between passes. This underscores the pivotal role of this parameter in influencing the surface texture. Furthermore, it is worth noting that the interaction effect of flow rate and path speed exerts a less pronounced influence on surface roughness in comparison to the other factors considered in our investigation.

### Validation study

In this empirical investigation, a set of regression equations is formulated to model and describe the behaviour of key material properties. The quadratic form of these equations is utilized to predict UTS, compressive strength, flexural strength, and impact strength, as articulated in Eqs. (1)–(5) correspondingly. These regression equations are grounded on the experimental responses and are contingent upon three pivotal predictor variables, specifically the spacing between passes, path speed, and flow rate.

1 $$ \begin{aligned}UTS \left[MPa\right] & = 41.2+ 97.6 A- 0.711 B + 1.68 C - 257 A*A + 0.0015 B*B \\ &\quad- 0.0853 C*C - 0.76 A*B - 0.81 A*C + 0.0565 B*C\end{aligned} $$

2 $$ \begin{aligned}Compressive strength \left[MPa\right]& = 77.78 + 157.0\mathrm{ A }+ 0.488\mathrm{ B }- 2.637\mathrm{ C }- 430.3\mathrm{ A}*\mathrm{A }- 0.00692\mathrm{ B}*\mathrm{B }\\ &\quad+ 0.0900\mathrm{ C}*\mathrm{C }- 0.765\mathrm{ A}*\mathrm{B }+ 0.12\mathrm{ A}*\mathrm{C }- 0.0041\mathrm{ B}*{\text{C}}\end{aligned} $$

3 $$ \begin{aligned}Flexural strength \left[MPa\right] & = 70.52 + 56.7\mathrm{ A}+ 0.550\mathrm{ B }+ 0.430\mathrm{ C }- 179.4\mathrm{ A}*\mathrm{A }- 0.01397\mathrm{ B}*\mathrm{B }\\ &\quad- 0.0187\mathrm{ C}*\mathrm{C }- 0.201\mathrm{ A}*\mathrm{B }+ 0.51\mathrm{ A}*\mathrm{C }+ 0.0038\mathrm{ B}*{\text{C}}\end{aligned} $$

4 $$ \begin{aligned}Impact strength \left[KJ/m\right] & = 10.60 + 6.62\mathrm{ A}- 0.0500\mathrm{ B}- 0.118\mathrm{ C }- 20.7\mathrm{ A}*\mathrm{A }- 0.00071\mathrm{ B}*\mathrm{B }- 0.00031\mathrm{ C}*\mathrm{C }\\ &\quad- 0.067\mathrm{ A}*\mathrm{B }- 0.001\mathrm{ A}*\mathrm{C }+ 0.00601\mathrm{ B}*{\text{C}}\end{aligned} $$

5 $$ \begin{aligned}Surface roughness \left[\mathrm{\mu m}\right] & = 3.86 + 20.39\mathrm{ A}- 0.0767\mathrm{ B }- 0.334\mathrm{ C }+ 2.86\mathrm{ A}*\mathrm{A }+ 0.002566\mathrm{ B}*\mathrm{B }\\ &\quad+ 0.01390\mathrm{ C}*\mathrm{C }- 0.1308\mathrm{ A}*\mathrm{B }- 0.387\mathrm{ A}*\mathrm{C }+ 0.00030\mathrm{ B}*{\text{C}}\end{aligned} $$

where A, B, and C denote spacing between passes, path speed, and flow rate, respectively.

Table 5 presents a concise comparison between experimental (E) and predicted (P) results for key response variables—UTS, compressive strength, flexural strength, impact strength, and surface roughness. This analysis provides valuable insights into the performance of our regression models. This comparative analysis is crucial for evaluating the predictive accuracy of our models, highlighting the complexities involved in predicting the mechanical properties and surface quality of MEX parts. For UTS, it is notable that the errors between experimental and predicted values exhibit both positive and negative discrepancies across various runs. This variability can be attributed to the intricate relationships between the selected ironing parameters and UTS. Runs such as 1, 4, and 8 experience errors exceeding 5%, highlighting the nuanced interplay of factors, including non-linear material responses and interactions, which our regression model seeks to capture. In the context of compressive strength, flexural strength, and impact strength, the comparative analysis demonstrates remarkably close agreement between experimental and predicted values, with errors generally falling within acceptable limits. These results affirm the effectiveness of our regression models in predicting these mechanical properties with a high degree of accuracy. Surface Roughness presents a distinct pattern with prediction errors varying across runs. Run 5 stands out with a 5.712% error, emphasizing the sensitivity of surface quality to specific parameter configurations. This discrepancy highlights the intricacies of predicting surface roughness and the need for careful consideration of interaction effects and non-linear responses in the model.

**Table 5:** Comparison between experimental (E) and predicted (P) results.

No	Responses														
	UTS			Compressive strength			Flexural strength			Impact strength			Surface roughness		
	(MPa)			(MPa)			(MPa)			(kJ/m^2^)			(µm)		
	E	P	Error (%)	E	P	Error (%)	E	P	Error (%)	E	P	Error (%)	E	P	Error (%)
1	51.16	53.86	− 5.28	72.70	72.86	− 0.22	81.44	81.89	− 0.55	9.46	9.48	− 0.21	2.993	2.875	3.94
2	49.77	48.87	− 1.19	68.22	68.65	− 0.63	79.91	80.02	− 0.14	9.14	9.01	1.42	5.682	5.759	− 1.36
3	55.36	56.26	− 1.63	74.74	74.31	0.58	82.57	82.46	0.13	9.45	9.58	− 1.38	3.302	3.225	2.33
4	50.93	48.24	5.28	67.20	67.05	0.22	80.23	79.78	0.56	8.86	8.84	0.23	5.468	5.585	− 2.14
5	54.56	51.95	4.78	76.94	76.56	0.49	83.68	83.12	0.67	9.81	9.63	1.83	2.731	2.887	− 5.71
6	45.25	46.24	− 2.19	71.37	70.72	0.91	80.54	80.33	0.26	9.07	9.03	0.44	5.935	5.896	0.66
7	54.60	53.61	1.81	75.83	76.48	− 0.86	82.88	83.09	− 0.25	9.52	9.56	− 0.42	3.357	3.396	− 1.16
8	43.68	46.29	− 5.98	70.49	70.87	− 0.54	80.77	81.33	− 0.69	8.77	8.95	− 2.05	5.786	5.630	2.70
9	54.29	54.20	0.17	76.86	77.08	− 0.29	82.12	82.23	− 0.13	9.62	9.78	− 1.66	4.629	4.591	0.82
10	47.73	49.44	− 3.58	76.61	77.42	− 1.06	81.35	82.01	− 0.81	9.09	9.15	− 0.66	4.727	4.648	1.67
11	51.11	49.40	3.35	78.34	77.53	1.03	83.00	82.33	0.81	9.16	9.11	0.55	4.603	4.682	− 1.72
12	55.85	55.94	− 0.16	77.26	77.04	0.28	82.99	82.88	0.13	9.84	9.67	1.73	4.762	4.800	− 0.80
13	52.30	54.23	− 3.69	76.77	75.71	1.38	83.11	84.23	− 1.35	9.63	9.51	1.25	4.086	4.076	0.24
14	53.92	54.23	− 0.57	76.47	75.71	0.99	85.73	84.23	1.75	9.76	9.51	2.56	4.148	4.076	1.74
15	56.47	54.23	3.97	73.89	75.71	− 2.46	83.85	84.23	− 0.45	9.13	9.51	− 4.16	3.994	4.076	-2.05

**Figure 5:**
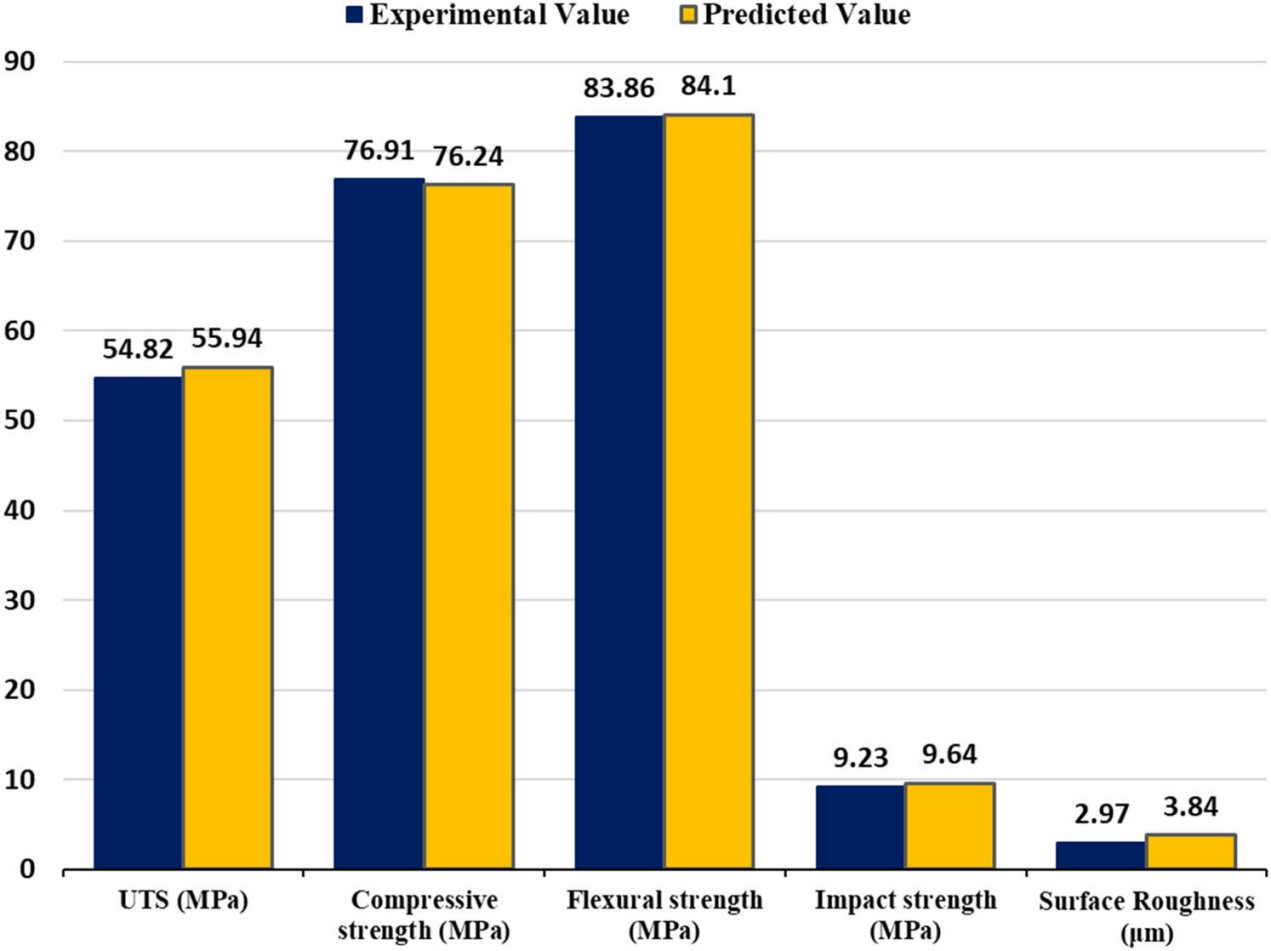
Validation analysis.

In conclusion, the experiment was conducted to determine the optimal ironing process parameters, with the spacing between passes set at 0.136113 mm, path speed at 23.5354 mm/s, and flow rate at 17.1717%. In the context of response optimizer, the composite desirability value signifies the overall optimization success in achieving the defined goals for multiple responses. The composite desirability considers how well the combination of variables satisfies the predefined objectives of multiple responses^77^. With a range of zero to one, where one is ideal, the obtained value suggests a high level of success in meeting the specified goals, as it is close to the ideal case^78^. In this study, the composite desirability of 0.811134 was achieved to maximize UTS, compressive strength, flexural strength, and impact strength while simultaneously minimizing surface roughness. The selection criteria for desirability were evaluated with equal weight and importance. The Multi-response prediction utilizing the response optimizer feature in Minitab software facilitated the identification of the optimum ironing process variables. The details of the desirability criteria selection are presented in Table 6. Subsequently, a validation analysis was performed to compare the optimized predicted response data with the optimized experimental response data. The analysis aimed to demonstrate the high degree of consistency between the regression equation and the experimental data, as illustrated in Fig. 5.

**Table 6:** Desirability criteria for responses.

Response	Goal	Lower	Target	Upper	Weight	Importance
UTS	Maximum	43.6834	56.4734		1	1
Compressive strength	Maximum	67.2000	78.3400		1	1
Flexural strength	Maximum	79.9100	85.7268		1	1
Impact strength	Maximum	8.7708	9.8390		1	1
Surface roughness	Minimum		2.7310	5.935	1	1

### Microscopic analysis of internal structure

The internal structure of MEX components is a crucial factor that determines their mechanical properties, failure mechanisms, and overall performance. The internal structure of a material refers to the arrangement of its atoms, molecules, and grains. It is responsible for the material’s strength, ductility, toughness, and other mechanical properties^79,80^. The internal structure also influences the material’s failure mechanisms, such as fatigue, creep, and fracture^81^. The internal structure of MEX components affects their thermal, electrical, and magnetic properties, which are essential for their performance in these applications^82,83^.

To visually depict the changes brought about by the ironing process, a series of distinct images were captured of the specimen's fracture section. These images serve to highlight the evolution of voids and layer bonding both before and after the application of the ironing process to every printed layer. Through this visual documentation, we elucidate the transformative impact of this post-processing technique on the internal structure of MEX components.

**Figure 6:**
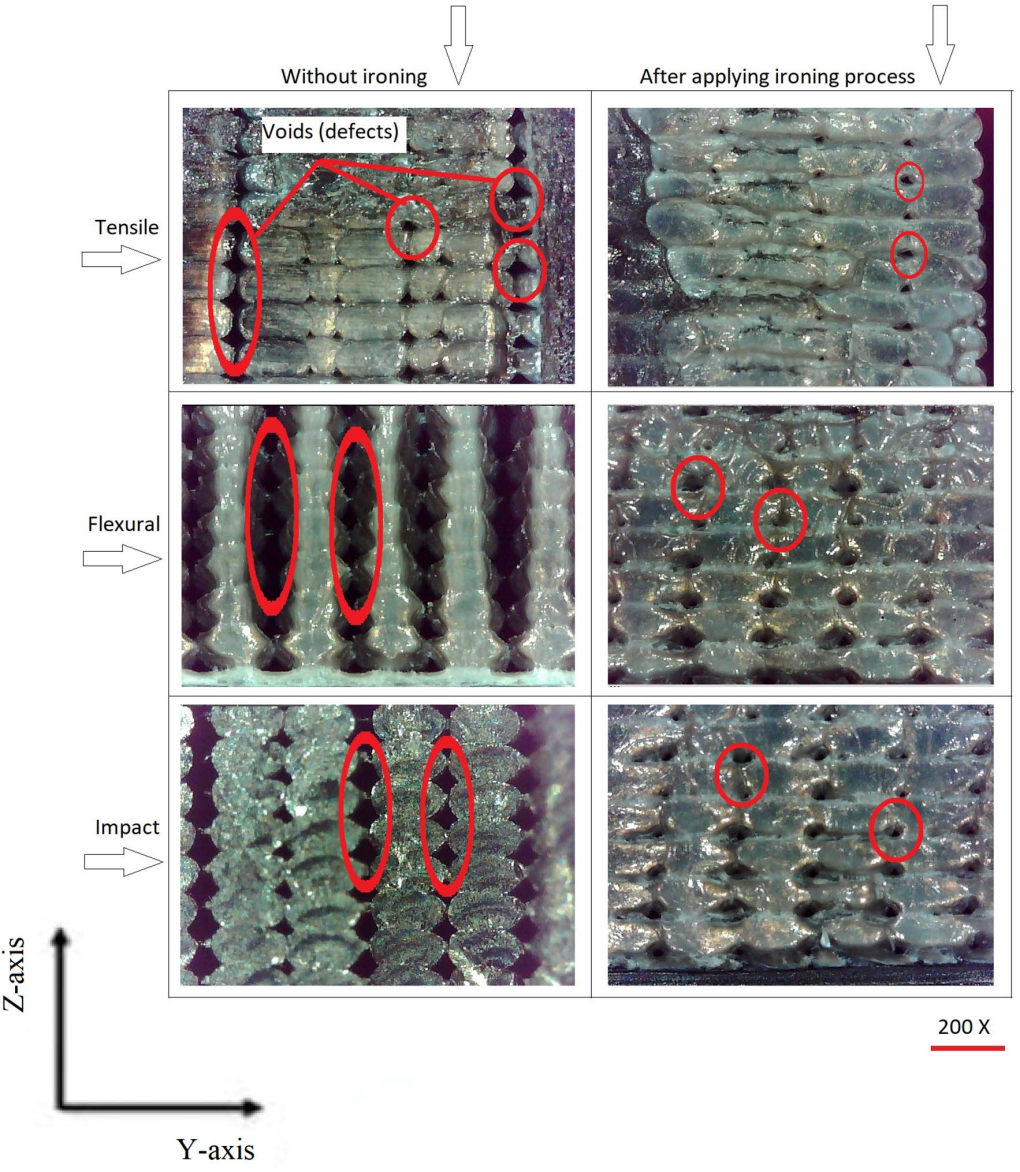
Micrographs of the cross-sectional area after fracture showing voids in the MEX parts.

**Figure 7:**
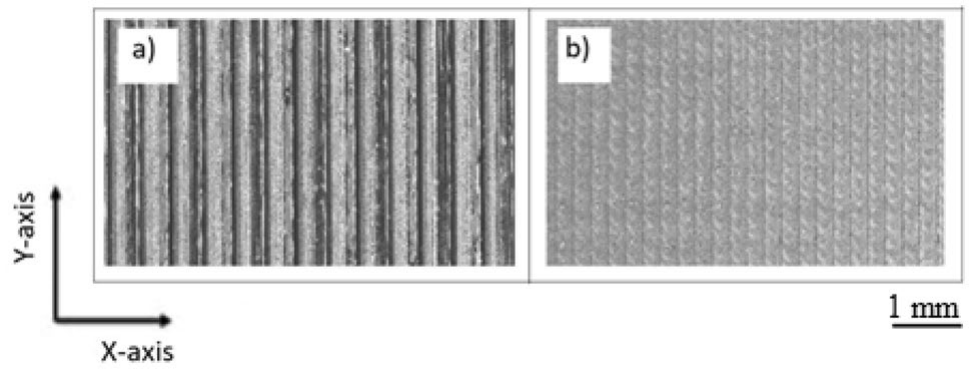
Top surface of the MEX parts: (**a**) before ironing, and (**b**) after ironing.

As shown in Figure 6, the conducted tests on the printed specimens revealed a noteworthy observation regarding the presence of voids within the material structure. The majority of these voids were observed to be concentrated in the region between the infill and the outer contour (wall) of the specimens and between the infill paths, signifying a critical area of interest for understanding material integrity and tensile strength. However, the transformative impact of the ironing process on the internal structure was remarkable. Following the application of the ironing technique, a substantial reduction in the visibility of voids was noted. These voids, once pronounced between the infill and wall, became considerably less discernible, indicating a significant enhancement in material cohesion and continuity. This reduction in void presence is indicative of improved layer bonding, a phenomenon that contributes to the substantial increase in tensile strength observed after the ironing process. The ironing process played a pivotal role in enhancing the surface roughness of the printed components. This enhancement was achieved through the substantial reduction of irregularities that were characteristic of the layer-by-layer deposition inherent to MEX technology, as can be seen in Fig. 7. As a result, the surface appeared notably smoother and more uniform, aligning with the criteria of aesthetics and functional performance. The marked improvement in surface roughness was closely tied to the reduction of irregularities between printed paths. Irregularities, characterized by discontinuities and variations in the surface, are often associated with Material Extrusion. Prior to the ironing process, these irregularities were a prominent feature of the printed parts, contributing to increased surface roughness. The ironing process's unique ability to level and smoothen these irregularities led to a substantial decrease in surface roughness, enhancing the overall quality of the printed components. Moreover, the ironing process played a pivotal role in minimizing voids within the material structure. These voids, initially present between printed paths, were considerably reduced in size and visibility after ironing. The process facilitated a closer and more cohesive bonding of adjacent material layers, reducing the voids that can compromise structural integrity, surface quality, and overall mechanical properties of MEX parts.

## Conclusion

The central objective of this investigation was to understand and optimize the intricate relationships between ironing process parameters and material properties in MEX technology. We boarded this journey with the aim of achieving not only enhanced mechanical properties but also improved surface quality, characteristics essential for diverse applications spanning various industries. The key findings of this study can be summarized as follows:

- The implementation of the ironing process in conjunction with the Box–Behnken Design BBD showcased a substantial improvement in the mechanical and surface properties of MEX-printed PLA samples.
- Optimization of ironing process parameters: through the application of DoE, specifically BBD, we identified optimal ironing process parameter combinations. This approach allowed us to systematically explore the parameter space and unveil the conditions that led to substantial enhancements in material properties.
- Effect of the ironing process: The introduction of the ironing process as a process step was a transformative aspect of our investigation. It significantly improved surface roughness, minimizing voids and enhancing layer bonding within the printed components. This enhancement was particularly evident in the results of tensile tests, where the presence of voids between infill and outer contour was drastically reduced. The surface roughness Ra experienced a substantial reduction, representing a decrease of approximately 69%.
- Mechanical property improvements: The integration of optimized ironing process parameters contributed to a remarkable increase in mechanical properties, as evidenced by higher ultimate tensile strength, compressive strength, flexural strength, and impact strength. The Ultimate Tensile Strength witnessed a noteworthy improvement of approximately 29%. Similarly, the compressive strength exhibited substantial growth, representing an enhancement of almost 25%. The flexural strength demonstrated a remarkable increase, signifying a considerable improvement of around 35%. Moreover, the impact strength saw a significant boost, showing an impressive improvement of about 162%. These improvements underscore the potential of this processing technique to elevate the overall mechanical performance of MEX-produced components.
- Surface quality enhancement: the ironing process not only heightened mechanical properties but also enhanced surface quality. Irregularities and imperfections were notably reduced, providing a smoother, more aesthetically pleasing surface finish.

In summary, this study explores the connections between ironing process parameters and material properties, offering insights into potential enhancements for the characteristics and surface quality of MEX-produced components. This research contributes to the ongoing efforts to optimize and advance MEX technology, emphasizing the impact of precision experimentation, post-processing techniques, and statistical methodologies in the pursuit of material innovation and the development of components with improved performance and aesthetics.

## Data Availability

The data that support the findings of this study are available on request from the corresponding author.
